# ENSEMBLE PLUS: final results of shorter ocrelizumab infusion from a randomized controlled trial

**DOI:** 10.1007/s00415-024-12326-z

**Published:** 2024-04-22

**Authors:** Hans-Peter Hartung, Thomas Berger, Robert A. Bermel, Bruno Brochet, William M. Carroll, Trygve Holmøy, Rana Karabudak, Joep Killestein, Carlos Nos, Francesco Patti, Amy Perrin Ross, Ludo Vanopdenbosch, Timothy Vollmer, Regine Buffels, Monika Garas, Karen Kadner, Marianna Manfrini, Qing Wang, Mark S. Freedman

**Affiliations:** 1 Department of Neurology, UKD, Medical Faculty, Heinrich-Heine University Düsseldorf, Düsseldorf, Germany; 2 Brain and Mind Centre, University of Sydney, Sydney, Australia; 3 Department of Neurology, Palacky University Olomouc, Olomouc, Czech Republic; 4 Department of Neurology, Medical University of Vienna, Vienna, Austria; 5 Comprehensive Center for Clinical Neurosciences and Mental Health, Medical University of Vienna, Vienna, Austria; 6 Mellen Center for MS, Neurological Institute, Cleveland Clinic, Cleveland, OH USA; 7 INSERM U 1215, Neurocentre Magendie, University of Bordeaux, Bordeaux, France; 8 Department of Neurology, Sir Charles Gairdner Hospital, Perron Institute for Neurological and Translational Science, The University of Western Australia, Nedlands, Australia; 9 Department of Neurology, Akershus University Hospital, Lørenskog, Norway; 10 Institute of Clinical Medicine, University of Oslo, Oslo, Norway; 11 Department of Neurology, Hacettepe University Faculty of Medicine, Ankara, Turkey; 12 Department of Neurology, MS Center Amsterdam, Amsterdam Neuroscience, Amsterdam UMC, Vrije Universiteit Amsterdam, Amsterdam, Netherlands; 13 Centre d’Esclerosi Múltiple de Catalunya (Cemcat), Vall d’Hebron Hospital Universitari, Barcelona, Spain; 14 Department of Medical and Surgical Sciences and Advanced Technologies, GF Ingrassia, UOS Sclerosi Multipla Policlinico G Rodolico, University of Catania, Catania, Italy; 15 Loyola University Chicago, Chicago, Maywood, IL USA; 16 Department of Neurology, AZ Sint Jan Brugge Oostende, Brugge, Belgium; 17 University of Colorado, Anschutz Medical Campus, Aurora, CO USA; 18 F. Hoffmann-La Roche Ltd, Basel, Switzerland; 19 Department of Medicine and the Ottawa Hospital Research Institute, University of Ottawa, Ottawa, ON Canada; 20 Basel, Switzerland

**Keywords:** Relapsing–remitting multiple sclerosis, Ocrelizumab, Shorter infusion, Infusion-related reaction, ENSEMBLE PLUS, Phase 3

## Abstract

**Introduction:**

Ocrelizumab is an approved intravenously administered anti-CD20 antibody for multiple sclerosis (MS). The safety profile and patient preference for conventional versus shorter ocrelizumab infusions were investigated in the ENSEMBLE PLUS study.

**Methods:**

ENSEMBLE PLUS was a randomized, double-blind substudy to the single-arm ENSEMBLE study (NCT03085810), comparing outcomes in patients with early-stage relapsing–remitting MS receiving ocrelizumab 600 mg over the approved 3.5-h (conventional) versus 2-h (shorter) infusion. The primary endpoint was the proportion of patients with infusion-related reactions (IRRs) following the first randomized dose (RD); the secondary endpoint included IRR frequency at subsequent RDs.

**Results:**

At first RD, the number of patients with an IRR in the conventional (101/373; 27.1%) versus shorter (107/372; 28.8%) infusion group was similar (difference, stratified estimates [95% CI]: 1.9% [− 4.4, 8.2]). Most IRRs (conventional: 99.4%; shorter: 97.7%) were mild/moderate. IRR frequency decreased over the course of RDs; three patients discontinued from the shorter infusion arm but continued with conventional infusion. Overall, > 98% of IRRs resolved without sequelae in both groups. Pre-randomization throat irritation was predictive of future throat irritation as an IRR symptom. Adverse events (AEs) and serious AEs were consistent with the known ocrelizumab safety profile. On completion of ENSEMBLE PLUS, most patients chose to remain on (95%) or switch to (80%) shorter infusion.

**Conclusion:**

ENSEMBLE PLUS demonstrates the safety and tolerability of shorter ocrelizumab infusions. Most patients remained on/switched to shorter infusion after unblinding; IRRs did not strongly influence patient decisions.

Clinical Trials Registration: Substudy of ENSEMBLE (NCT03085810). Registration: March 21, 2017.

**Supplementary Information:**

The online version contains supplementary material available at 10.1007/s00415-024-12326-z.

## Introduction

Ocrelizumab (OCR) is a humanized monoclonal antibody that selectively targets CD20+ B cells. It is approved for the treatment of relapsing multiple sclerosis (MS) and primary progressive MS [1, 2]. In the pivotal phase 3 trials of OCR (OPERA I [NCT01247324], OPERA II [NCT01412333], and ORATORIO [NCT01194570]), infusion-related reactions (IRRs) were one of the most common adverse events (AEs) [3, 4]. When pooling the data from OPERA and ORATORIO, it was observed that IRRs were mostly mild to moderate, were more frequent with the first OCR infusion, and decreased with subsequent infusions [4, 5]. The current administration of OCR begins with an initial dose of two 300 mg intravenous (IV) infusions, given 2 weeks apart and each lasting at least 2.5 h. Subsequent doses are administered as single 600 mg infusions every 6 months and last at least 3.5 h [1, 2]. In addition, premedication is given 30–60 min prior to each infusion of OCR, and patients are observed for 1 h following infusion [1, 2]. Reducing the infusion time and therefore minimizing the time required to attend the infusion site may reduce the treatment burden for patients, their caregivers, and hospital staff; however, it should be done without negatively affecting patient safety [6–8].

The safety of a shorter infusion of OCR has been investigated in ENSEMBLE PLUS—a randomized, double-blind substudy to the single-arm ENSEMBLE study (NCT03085810). A shorter infusion of OCR was compared with conventional infusion in a subgroup of eligible patients with relapsing–remitting MS (RRMS) enrolled in the main ENSEMBLE study. The proportion of patients with an IRR during infusion or within 24 h following the first randomized dose (RD) was used as a primary endpoint. Results from the full cohort of 745 patients randomized into ENSEMBLE PLUS (interim clinical cut-off date [CCOD]: December 13, 2019) demonstrated that the rates and severity of IRRs were similar between conventional and shorter OCR infusion periods, following the first RD [9].

Here, we describe the results from the full cohort of patients (*N* = 745) in ENSEMBLE PLUS, who received between one and six RDs up to the CCOD of March 4, 2022. The primary endpoint, as described above, has been previously reported by Hartung et al. [9]. Secondary endpoints included the proportion of patients with IRRs overall, proportion of patients with IRRs by dose at and after randomization, severity and symptoms of IRRs, IRRs leading to treatment discontinuation, and overall safety. Exploratory endpoints included the number of, and proportion of patients with, Grade 3 (severe) or Grade 4 (life-threatening) IRRs as defined by the Common Terminology Criteria for Adverse Events (CTCAE) and the predictivity of IRR symptoms for future IRRs. The proportion of patients choosing to remain on or switch to the shorter infusion protocol is also reported.

## Methods

### Study design and patients

ENSEMBLE PLUS is a phase 3b prospective, multicenter, randomized, double-blind substudy designed to evaluate the safety of a shorter infusion of 2 h versus the conventional 3.5-h infusion of OCR in patients with early-stage RRMS enrolled in the main ENSEMBLE study. Detailed study methodology has been reported previously and is summarized here [9].

The population of the substudy was based on the eligibility criteria for the main ENSEMBLE study, and as prespecified in the protocol, the primary endpoint was assessed in the intent-to-treat (ITT) population. Since the primary analysis (CCOD: September 27, 2019; *N* = 580) [9], the study continued to recruit more patients, up to 373 and 372 patients in the conventional and shorter arms, respectively (*N* = 745). All patients were followed up for a period of 48 weeks after the last infusion of the study drug (CCOD: March 4, 2022). Eligible patients were treatment-naive, aged 18–55 years, had a confirmed diagnosis of RRMS (as per 2010 McDonald criteria) [10], a disease duration ≤ 3 years, one or more relapses/signs of MRI activity in the prior 12 months, and an Expanded Disability Status Scale score of 0–3.5 (inclusive) at screening. Patients were excluded from the substudy if they had experienced any previous serious IRRs with OCR. The substudy enrollment target was 700 patients, to comprise 150 patients already participating in the main ENSEMBLE study and a further 550 newly enrolled patients. The first patient enrollment in the ENSEMBLE substudy (for patients already enrolled in the main study) was on November 1, 2018; the first newly enrolled patient was randomized into the substudy on March 22, 2019.

OCR 600 mg infusions were given every 24 weeks, with mandatory premedication to reduce the severity and frequency of IRRs: The majority of patients received 100 mg IV methylprednisolone or its equivalent approximately 30 min prior to each OCR infusion, and antihistamines approximately 60 min before every infusion. All patients were given the first dose of OCR as per the label—two 300 mg infusions, separated by 14 days. Patients were randomized 1:1 to either the conventional infusion group or the shorter infusion group. Randomization occurred at Week 24 for newly enrolled patients, and at the next scheduled infusion visit for patients already participating in the main ENSEMBLE study (Week 48, 72, 96, or 120). Randomization of all patients was stratified by region (USA/Canada/Australia versus rest of the world), and by dose at which the patient was randomized in the main study (ENSEMBLE participants only). Patients in the conventional infusion group received a 3.5-h infusion of 600 mg OCR in 500 mL 0.9% sodium chloride every 24 weeks until the study end. Patients in the shorter infusion group received a 2-h infusion of 600 mg OCR in 500 mL 0.9% sodium chloride, followed by a 1.5-h infusion of 100 mL 0.9% sodium chloride to mimic the conventional infusion duration of 3.5 h, every 24 weeks until the study end. Blinding of patients, site personnel, and the sponsor study management team was maintained throughout the study. Preloaded infusions were covered with infusion cover bags at the pharmacy. An unblinded infusion nurse managed the preloaded infusions, operated the administration pump, and recorded the infusion times. A clinical nurse monitored patient vital signs, clinical status, tolerance, and safety of the patient during infusion, managing patients with the appropriate actions, in collaboration with the infusion nurse. Following the unblinding of the ENSEMBLE PLUS study, patient preference for either conventional or shorter infusion was assessed. This manuscript reports results from the CCOD of March 4, 2022.

### Sample collection

The collection of pharmacokinetic (PK) blood samples occurred at the first post-randomization OCR infusion only. Three samples were taken at different time points throughout the infusion: (1) The pre-infusion sample was taken 5–30 min before the IV methylprednisolone premedication; (2) a sample was taken at 30 min after the switch/mimic switch for the peak concentration of the shorter infusion; (3) and a post-infusion sample was taken 30 min after the completion of the OCR infusion, representing the peak concentration of the conventional infusion of OCR.

PK measurements were taken only at the first RD. The PK analysis assessed the OCR maximum concentration (*C*_max_), and any potential relationship between the primary endpoint and *C*_max_. PK data were analyzed based on the PK population, defined as all randomized patients receiving any OCR treatment who had ≥ 1 measurable concentration value.

### Method of treatment assignment

Patients were randomized into two groups (conventional infusion or shorter infusion group) in a 1:1 ratio. An independent voice/Web response system (IxRS) provider conducted randomization (with the use of blocked randomization). For patients previously enrolled on the main ENSEMBLE study, randomization took place at the next scheduled infusion visit (i.e., at Dose 3, 4, 5, or 6). For newly enrolled patients, randomization took place at Dose 2. Patients were stratified by region (USA/Canada/Australia versus rest of the world) and the dose at which the patient received their first RD.

### Safety reporting

Safety assessments consisted of monitoring and recording AEs and serious AEs from the first RD onwards until the CCOD, even if the onset was after the patient discontinued randomized treatment. AEs were defined as all AEs including IRRs and serious MS relapses but excluded nonserious MS relapses. Serious AEs were defined as all serious AEs, including serious MS relapses and serious IRRs. AEs and serious AEs were coded using Medical Dictionary for Regulatory Activities (MedDRA) Version 24.1 and summarized by System Organ Class (SOC) and preferred term (PT). The safety population included all randomized patients who received any dose or part of a dose of OCR treatment. All safety assessments were evaluated in the safety population, and patients were analyzed according to the actual treatment received.

An AE electronic Case Report Form (eCRF) was used to capture IRRs, defined as any AE that occurred during infusion or within 24 h after the infusion. Patients were also contacted via phone after 24 h post-infusion to capture any other IRR that might have occurred during this time period. Clinical and vital signs related to IRR symptoms during infusion were collected by a blinded nurse. IRR symptoms were reported on the dedicated IRR eCRF and were coded by MedDRA and summarized by SOC and PT. Any IRR event occurring in a patient both during and 24 h post-infusion was reported as per protocol; multiple IRRs in one patient are counted only once at the most extreme (highest) intensity observed. Any IRR event occurring in patients after 24 h post-infusion was reported as a general AE. All randomized patients were included in the ITT population. IRRs were evaluated in the ITT population, and patients were analyzed according to their randomized treatment, regardless of the treatment actually received.

### Statistical methods

The sample size is expected to provide sufficient precision around the IRR rates for this substudy. The pooled IRR rate across all doses excluding Dose 1 is expected to be 12% in the substudy, based on the observed IRR rate at each dose in the OPERA studies [5]. The primary endpoint was stratified by the region and first RD. The weighted average of the proportion difference between the two randomized groups across strata was estimated using the Cochran–Mantel–Haenszel method. The associated 95% confidence interval (CI) was obtained using the normal approximation method. The unstratified difference in the proportions between the two randomized groups was also presented along with an associated 95% CI. This summary was presented for all patients, for patients with at least one prefirst RD IRR, and for patients with no prefirst RD IRRs. Descriptive statistics were used to summarize safety assessments, with no formal hypothesis testing. A box plot was used to show the relationship between OCR *C*_max_ versus IRR maximum intensity (none, mild, moderate, severe, life-threatening, and fatal) at first RD, for the conventional infusion and the shorter infusion groups.

### Standard protocol approvals, registrations, and patient consent

The trial protocols (NCT03085810) were approved by the relevant institutional review boards/ethics committees, and written informed consent was provided by all patients. The trial data were collected by the Steering Committee and study investigators and analyzed by the study sponsor. Submission of the manuscript publication was agreed upon by the authors and the Steering Committee.

## Results

### Patient disposition and analysis population

**Fig. 1:**
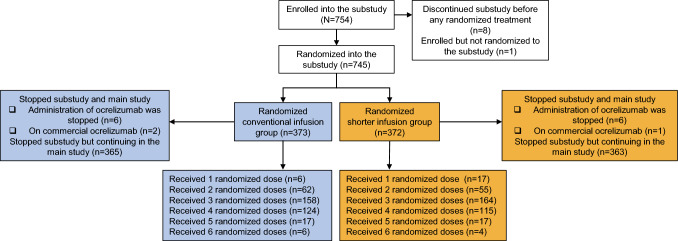
Patient disposition and analysis population. Of the 754 patients enrolled, eight patients (all newly enrolled) consented to participate in the ENSEMBLE PLUS substudy but were never randomized and one patient (already enrolled) in the main ENSEMBLE study was never randomized into the ENSEMBLE PLUS substudy. Patients (*N* = 745) were randomized in a 1:1 ratio to the conventional infusion group (*n* = 373) or the shorter infusion group (*n* = 372). Of the 373 patients from the conventional infusion group who discontinued randomized treatment, six patients stopped the substudy and main study, and in turn, administration of OCR was stopped; two patients stopped the substudy and main study but continued on commercial OCR; and 365 patients stopped the substudy but continued in the main study. Of the 372 patients from the shorter infusion group who discontinued randomized treatment, six patients stopped the substudy and main study, and in turn, administration of OCR was stopped; one patient stopped the substudy and main study but continued on commercial OCR; and 363 patients stopped the substudy but continued in the main study. *OCR* ocrelizumab

The ENSEMBLE PLUS study enrolled a total of 754 patients (207 from the main ENSEMBLE study and 547 newly enrolled patients) at 97 investigational sites across 22 countries. Of these, 745 patients were randomized (1:1) to the conventional infusion group (*n* = 373) or shorter infusion group (*n* = 372) (Fig. 1).

Baseline demographics and disease characteristics were well balanced across both infusion groups, as previously reported (Table 1) [9].

**Table 1:** Baseline patient demographics and disease characteristics

	Conventional infusion (*N* = 373)	Shorter infusion (*N* = 372)
Age at first randomized dose^a^, years, mean (SD)	34.2 (8.6)	34.2 (9.0)
Sex, *N* (%)		
Male	138 (37.0)	133 (35.8)
Female	235 (63.0)	239 (64.2)
Race, *N* (%)		
African Indian or Alaskan Native	2 (0.5)	4 (1.1)
Asian	4 (1.1)	5 (1.3)
Black or African American	15 (4.0)	11 (3.0)
Native Hawaiian or other Pacific Islander	0 (0.0)	1 (0.3)
White	312 (83.6)	308 (82.8)
Multiple	16 (4.3)	20 (5.4)
Unknown	24 (6.4)	23 (6.2)
Weight, kg, mean (SD)	76.7 (20.1)	75.5 (21.1)
BMI, kg/m^2^, mean (SD)	26.3 (6.2)	26.1 (6.7)
Time since the first symptom^a^, years, mean (SD)	1.8 (1.0)	1.8 (1.2)
Number of patients with previous IRR, *N* (%)	108 (29.0)	115 (30.9)
Randomization assignment by stratification, *N*		
USA/Canada/Australia	112	112
RoW	261	260

All patients, ITT population. With the exception of age and duration since RMS diagnosis, all other demographic characteristics were recorded at the screening visit of the ENSEMBLE study

*BMI* body mass index, *IRR* infusion-related reaction, *ITT* intent-to-treat, *MS* multiple sclerosis, *RD* randomized dose, *RMS* relapsing multiple sclerosis, *RoW* rest of the world, *SD* standard deviation

^a^Calculated as the date of first RD minus date of birth (for age) or date of first MS symptom (for time since first symptom), divided by 365.25

As of CCOD (March 4, 2022), no patients were withdrawn from the main ENSEMBLE study due to IRRs. However, three patients (0.8%) were discontinued early from the substudy due to IRRs in the shorter infusion group; two of these patients continued with OCR treatment in the main study, and one patient was recorded as lost to follow-up. Non-safety reasons for early discontinuation from the substudy in the conventional and shorter infusion groups, respectively, were subject consent withdrawn (*n* = 5 [1.3%], *n* = 7 [1.9%]), other (*n* = 13 [3.5%], *n* = 8 [2.2%]), and substudy terminated by the sponsor (*n* = 355 [95.2%], *n* = 352 [95.1%]). After termination of the substudy, 98% of patients continued OCR treatment in the main ENSEMBLE study.

### Dosing and infusion times

All patients (*N* = 745) received at least one RD of OCR. In the conventional and shorter arms, the majority of patients received three to four RDs, 75.6% (*n* = 282/373) and 75.0% (*n* = 279/372), respectively (Fig. 1). Across all RDs, the median (interquartile range) infusion times were 215 (210–224) min in the conventional infusion group and 120 (120–130) min in the shorter infusion group, resulting in a reduction in median infusion time of approximately 95 min.

### Infusion-related reactions

**Fig. 2:**
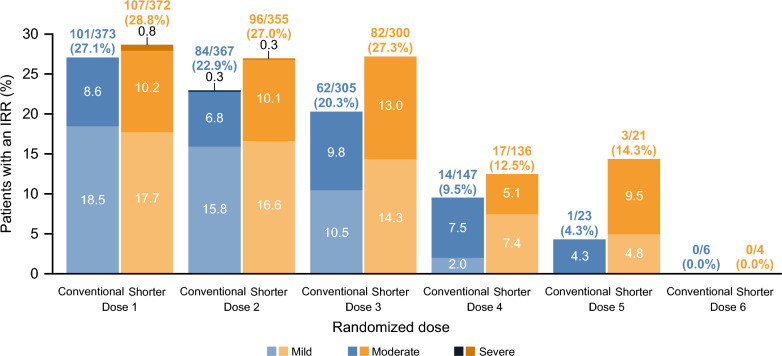
IRRs by RD and severity (ITT). Percentages for the number of patients with an infusion are based on *N*, and percentages for the number of patients with any IRR are based on number of patients with an infusion. Percentages of patients with IRR grade are based on number of patients with any IRR. For summaries by grade, multiple IRRs in one patient are counted only once at the most extreme (highest) intensity observed. *IRR* infusion-related reaction, *ITT* intent-to-treat, *RD* randomized dose

A summary of IRRs that occurred at all RDs is presented in Fig. 2. As previously reported, the number of patients with IRRs prior to the first RD was *n* = 108 (29.0%) in the conventional and *n* = 115 (30.9%) in the shorter infusion groups. In both arms, IRRs were most frequently experienced at the first RD and decreased in frequency thereafter. Additionally, there were no clinically meaningful changes from the mean pre-infusion baseline values in vital signs at first RD (pulse rate, systolic and diastolic blood pressure) or between infusion groups over time. Median peak OCR concentrations (*C*_max_) were comparable between infusion groups at first RD (conventional: 200.5 μg/mL versus shorter: 202.0 μg/mL, consistent with previously reported *C*_max_ values and did not correlate with observed IRRs) (Supplementary Fig. 1). IRRs at first RD were treated with paracetamol (22.7% and 13.0%), diphenhydramine hydrochloride (20.5% and 28.3%), and chlorphenamine (18.2% and 19.6%) in the conventional and shorter infusion groups, respectively.

The number of patients who experienced an IRR (during or within 24 h post-infusion) following the first RD (primary endpoint) was similar between the conventional and shorter infusion groups (101/373 patients [27.1%] vs 107/372 patients [28.8%]; difference, stratified estimates [95% CI]: 1.9% [− 4.4, 8.2]; Fig. 2). The proportion of patients experiencing IRRs at the second, third, fourth, fifth, and sixth RDs was 22.9%, 20.3%, 9.5%, 4.3%, and 0.0% in the conventional infusion group and 27.0%, 27.3%, 12.5%, 14.3%, and 0.0% in the shorter infusion group, respectively (Fig. 2). Across all RDs, the proportion of patients with any IRRs was similar between conventional and shorter infusion groups (41.6% and 46.2%, respectively). One serious IRR (Grade 2 IRR characterized by Grade 2 cough and oropharyngeal pain) occurred at the second RD in the shorter infusion group. The IRR was resolved but was considered “serious” due to hospitalization for observation.

**Fig. 3:**
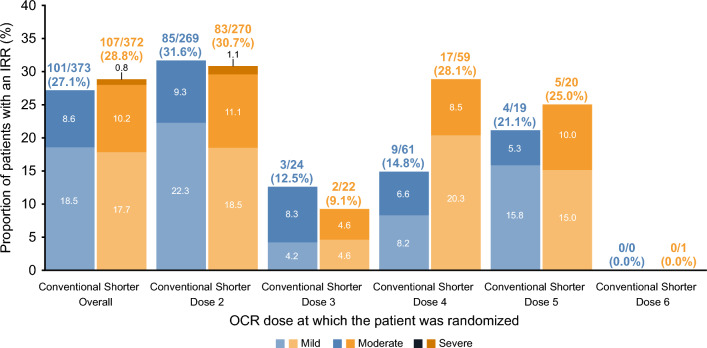
IRRs at the first RD by OCR dose at which the patient was randomized. Percentages are based on *N*, number of patients that received the infusion at the OCR dose at which the patient was randomized. All IRRs are included in this graph, both serious and nonserious. *Mild* mild (Grade 1), *Mod.* moderate (Grade 2), *Sev.* severe (Grade 3), *L-T* life-threatening (Grade 4), *Fatal* fatal (Grade 5). *IRR* infusion-related reaction, *OCR* ocrelizumab, *RD* randomized dose

The majority of IRRs across all RDs were mild or moderate (Grade 1 or Grade 2) (99.4% in the conventional infusion group and 97.7% in the shorter infusion group) (Fig. 2), and there were five severe (Grade 3) IRRs in total; one in the conventional infusion group (laryngeal inflammation) and four in the shorter infusion group (headache, oropharyngeal pain/pharyngeal swelling, fatigue, hypertension, and throat irritation). No patients experienced an IRR at or above Grade 3 after the second RD. No IRRs were life-threatening, fatal, or resulted in discontinuation from ENSEMBLE. IRR outcomes were generally favorable with most IRRs reported as recovered or resolved in the conventional (≥ 98.4%) and shorter infusion groups (≥ 97.9%) across all RDs. In patients receiving the first RD at Dose 4, 5, or 6, the rate of IRRs at first RD was lower in the conventional infusion group (*n* = 13/80; 16.3%) compared with the shorter infusion group (*n* = 22/80; 27.5%) (Fig. 3).

The most frequent IRR symptoms at first RD in the conventional and shorter infusion groups, respectively, were throat irritation (18.8%; 29.9%) and dysphagia (6.9%; 7.5%) during the infusion, and headache (25.7%; 17.8%) and fatigue (22.8%; 18.7%) within 24 h post-infusion (Tables 2 and 3).

**Table 2:** IRR symptoms during first RD (IRR during OCR/saline infusion)

	Conventional infusion (*N* = 373)	Shorter infusion (*N* = 372)
Number (%) of patients with an infusion	373 (100.0)	372 (100.0)
Number (%) of patients with any IRR	101 (27.1)	107 (28.8)
Number (%) of patients with any IRR during the OCR/saline infusion	44/101 (43.6)	65/107 (60.7)
Respiratory, thoracic, and mediastinal disorders^a^	24/101 (23.8)	40/107 (37.4)
Throat irritation	19/101 (18.8)	32/107 (29.9)
Oropharyngeal pain	4/101 (4.0)	6/107 (5.6)
Gastrointestinal disorders^a^	9/101 (8.9)	11/107 (10.3)
Dysphagia	7/101 (6.9)	8/107 (7.5)
Skin and subcutaneous tissue disorders^a^	7/101 (6.9)	10/107 (9.3)
Rash	1/101 (1.0)	7/107 (6.5)
Ear and labyrinth disorders^a^	6/101 (5.9)	7/107 (6.5)
Ear pruritus	6/101 (5.9)	6/107 (5.6)
General disorders and administration site conditions^a^	6/101 (5.9)	2/107 (1.9)

Percentages for the number of patients with an infusion are based on *N*, and percentages for the number of patients with any IRR are based on number of patients with an infusion. Percentages of patients with symptoms within each time period (e.g., “During OCR/saline infusion”) are based on number of patients with any IRR. Percentages of patients with any symptoms are based on number of patients with any IRR. IRR symptoms are displayed in descending order of frequency of System Organ Class and by preferred term within System Organ Class. If a patient experiences more than one episode of an IRR symptom, then the patient is counted only once for that symptom. If a patient has more than one symptom in a System Organ Class, then the patient is counted only once in that System Organ Class. System Organ Class and preferred terms were defined using MedDRA Version 24.1 thesaurus terms

*IRR* infusion-related reaction, *MedDRA* Medical Dictionary for Regulatory Activities, *OCR* ocrelizumab, *RD* randomized dose

^a^Only IRRs occur in ≥ 5% of patients in at least one treatment group

**Table 3:** IRR symptoms during first RD (IRR with 24 h after end of OCR/saline infusion)

	Conventional infusion (*N* = 373)	Shorter infusion (*N* = 372)
Number (%) of patients with any IRR within 24 h after the end of the OCR/saline infusion	68/101 (67.3)	54/107 (50.5)
Nervous system disorders^a^	34/101 (33.7)	22/107 (20.6)
Headache	26/101 (25.7)	19/107 (17.8)
Dizziness	4/101 (4.0)	1/107 (0.9)
General disorders and administration site conditions^a^	31/101 (30.7)	22/107 (20.6)
Fatigue	23/101 (22.8)	20/107 (18.7)
Pyrexia	4/101 (4.0)	1/107 (0.9)
Gastrointestinal disorders^a^	9/101 (8.9)	11/107 (10.3)
Nausea	8/101 (7.9)	7/107 (6.5)
Respiratory, thoracic, and mediastinal disorders^a^	11/101 (10.9)	5/107 (4.7)
Vascular disorders^a^	13/101 (12.9)	3/107 (2.8)
Flushing	9/101 (8.9)	3/107 (2.8)
Musculoskeletal and connective tissue disorders^a^	7/101 (6.9)	4/107 (3.7)
Skin and subcutaneous tissue disorders^a^	5/101 (5.0)	3/107 (2.8)

Percentages for the number of patients with an infusion are based on *N*, and percentages for the number of patients with any IRR are based on number of patients with an infusion. Percentages of patients with symptoms within each time period (e.g., “During OCR/saline infusion”) are based on number of patients with any IRR. Percentages of patients with any symptoms are based on number of patients with any IRR. IRR symptoms are displayed in descending order of frequency of System Organ Class and by preferred term within System Organ Class. If a patient experiences more than one episode of an IRR symptom, then the patient is counted only once for that symptom. If a patient has more than one symptom in a System Organ Class, then the patient is counted only once in that System Organ Class. System Organ Class and preferred terms were defined using MedDRA Version 24.1 thesaurus terms

*IRR* infusion-related reaction, *MedDRA* Medical Dictionary for Regulatory Activities, *OCR* ocrelizumab, *RD* randomized dose

^a^Only IRRs occur in ≥ 5% of patients in at least one treatment group

**Fig. 4:**
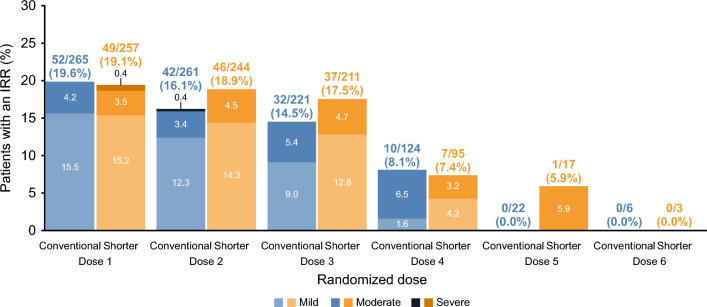
IRRs by RD and severity (patients without prefirst RD IRR, ITT). Percentages for the number of patients with an infusion are based on *N*, and percentages for a number of patients with any IRR are based on number of patients with an infusion. *IRR* infusion-related reaction, *ITT* intent-to-treat, *RD* randomized dose

In both infusion groups, IRRs occurred with a higher incidence in patients with at least one prefirst RD IRR (conventional infusion group [45.4%] and shorter infusion group [50.4%]) compared with those with no prefirst RD IRR (conventional infusion group [19.6%] and shorter infusion group [19.1%]) (Fig. 4).

In patients randomized at Dose 3, 4, 5, or 6 (from the already enrolled patients), the percentage of patients with prefirst RD IRRs was higher in the shorter infusion group (37.3%) versus the conventional infusion group (25.0%). Across all RDs, 39/373 patients (10.5%) and 55/372 patients (14.8%) had an IRR that led to intervention (i.e., slowing down or temporary interruption of the infusion) in the conventional and shorter infusion groups, respectively. Patients with ≥ 1 prefirst RD IRR of throat irritation were more likely to experience IRRs at all RDs (66.7% and 69.6% of patients in the conventional and shorter infusion groups, respectively) compared with patients who had no prefirst RD throat irritation (31.3% and 35.8% of patients in the conventional and shorter infusion groups, respectively). All cases of throat irritation during the infusion were Grade 1 or Grade 2, and no patients discontinued the infusion due to throat irritation. The proportion of patients who experienced headache as an IRR symptom was similar in subjects with a medical history of migraine or headache (20.4% and 17.8% in the conventional and shorter infusion groups, respectively) and subjects with no medical history of migraine or headache (15.4% and 15.3% in the conventional and shorter infusion groups, respectively).

### Adverse events reported in the safety population

Overall, 79.5% of patients (*n* = 294/370) in the conventional infusion group and 81.9% of patients (*n* = 307/375) in the shorter infusion group experienced AEs (Table 4). The most common AEs, reported in ≥ 5% of patients in each treatment group, were IRRs (conventional: 41.5%; shorter: 46.1%), nasopharyngitis (conventional: 15.7%; shorter: 13.3%), and headache (conventional: 12.7%; shorter: 9.9%) (Table 4). Most AEs were mild or moderate with serious AEs reported in 5.7% and 5.1% of patients from the conventional and shorter infusion arms, respectively (Table 4); however, no serious AEs led to treatment withdrawal in either group. Grade 4 (life-threatening) AEs were reported in four patients (typhoid fever, suicidal ideation, neutrophil count decreased, and ectopic pregnancy) in the conventional infusion group and in two patients (neutropenia and febrile neutropenia) in the shorter infusion group (Table 4). Fatal AEs were reported in three patients, all of whom were COVID-19–related (two patients in the conventional arm and one patient in the shorter arm). AEs leading to temporary interruption of OCR treatment occurred in three (0.8%) patients in both the conventional and shorter infusion groups, respectively (Table 4). There were no AEs leading to treatment withdrawal of the patients from the conventional infusion group and two patients (0.5%) from the shorter infusion group withdrew from OCR treatment due to an AE (serous retinopathy and peripheral edema).

**Table 4:** Summary of treatment-emergent adverse events

	Conventional infusion (*N* = 370)	Shorter infusion (*N* = 375)
Total number (%) of patients with ≥ 1 AE	294 (79.5)	307 (81.9)
Total number of AEs	1482	1411
AEs by severity, *N* of patients		
Grade 1	66	71
Grade 2	201	212
Grade 3	21	21
Grade 4	4	2
Grade 5	2	1
Total number (%) of deaths	2 (0.5)	1 (0.3)
Total number (%) of patients with ≥ 1		
AE with fatal outcome	2 (0.5)	1 (0.3)
Serious AE^a^	21 (5.7)	19 (5.1)
Serious AE leading to withdrawal from OCR treatment	0 (0.0)	0 (0.0)
Serious AE leading to OCR temporary dose interruption^b^	1 (0.3)	0 (0.0)
AE leading to withdrawal from OCR treatment	0 (0.0)	2 (0.5)
AE leading to OCR temporary dose interruption^b^	3 (0.8)	3 (0.8)
IRRs leading to withdrawal from OCR treatment at the first RD	0 (0.0)	0 (0.0)
IRRs leading to withdrawal from OCR treatment at any RD	0 (0.0)	0 (0.0)
Medical concepts: patients (%) with		
Malignancies^c^	0 (0.0)	1 (0.3)
Infections	158 (42.7)	169 (45.1)
Serious infections^d^	6 (1.6)	10 (2.7)
Total number (%) of patients with ≥ 1 AE occurring at relative frequency ≥ 5%^e^	218 (58.9)	239 (63.7)
IRR	154 (41.6)	173 (46.1)
Nasopharyngitis	58 (15.7)	50 (13.3)
Headache	47 (12.7)	37 (9.9)
Upper respiratory tract infection	28 (7.6)	35 (9.3)
Fatigue	28 (7.6)	28 (7.5)
UTI	20 (5.4)	25 (6.7)
Paraesthesia	20 (5.4)	15 (4.0)

Investigator text for AEs is coded using MedDRA Version 24.1. Percentages are based on *N* in the column headings. Multiple occurrences of the same AE in an individual are counted only once except for “Total number of AEs” row in which multiple occurrences of the same AE are counted separately. Treatment-emergent AEs (i.e., “First RD”-emergent AEs) are defined as either: (a) AEs with an observed or imputed date of AE onset, which is on or after the date of first RD; or (b), AEs with an observed or imputed date of AE onset, which is before the date of first RD and which later worsens in intensity. Non serious relapses are excluded

*AE* adverse event, *CRF* Case Report Form, *eCRF* electronic Case Report Form, *IRR* infusion-related reaction, *MedDRA* Medical Dictionary for Regulatory Activities, *MS* multiple sclerosis, *OCR* ocrelizumab, *RD* randomized dose, *SAE* serious adverse event, *UTI* urinary tract infection

^a^Grade 4 (life-threatening) AEs were reported in four patients (typhoid fever, suicidal ideation, neutrophil count decreased, and ectopic pregnancy) in the conventional infusion group and two patients (neutropenia and febrile neutropenia) in the shorter infusion group. Grade 5 (fatal) AEs of COVID-19 were reported in two patients in the conventional infusion group and COVID-19 pneumonia in one patient in the shorter infusion group

^b^Based on the Adverse Event/IRR/MS Relapse eCRF page, where the response to the question “Action taken with OCR due to SAE/AE” is “Temporary dose interruption” or “Dose delayed”

^c^Malignancies are identified using AEs falling into the Standard MedDRA Query “Malignant tumors (narrow)”

^d^Serious infections are defined using AEs falling into the MedDRA System Organ Class “Infections and Infestations” and using “Is the event nonserious or serious?” from the Adverse Events CRF page

^e^Only AEs occur in ≥ 5% of patients in at least one treatment group

### After unblinding

After unblinding the study, 20.3% (*n* = 71/350) of patients in the conventional infusion group chose to stay on the conventional infusion and 79.7% (*n* = 279/350) of patients chose to switch to shorter infusions. From the shorter infusion group, 5.4% (*n* = 19/350) of patients chose to switch to the conventional infusion with the majority (94.6%; *n* = 331/350) of patients choosing to continue with the shorter infusion protocol. Of the patients who chose conventional infusion after unblinding, 57.7% (*n* = 51/90) had experienced IRRs during the blinded study period compared with 42.0% (*n* = 256/610) of patients who chose shorter infusion.

### Limitations

A limitation of this substudy was the lack of formal statistical testing, as only descriptive statistics were provided for differences in IRR severity, IRR type, and AEs between the two arms. A significant limitation when analyzing IRR frequency across RDs relates to the relatively low number of patients who completed later doses in this study, making the results less interpretable for the later doses. For example, a notably high proportion of patients reported IRRs at the third RD in the shorter infusion arm compared with conventional infusion. However, upon closer inspection this difference was largely driven by the fact that more patients with pre-randomization IRRs were randomized to the shorter infusion arm and because IRR frequency at the fifth dose had very low patient numbers in both arms, thereby increasing the probability of overestimating the difference between treatment arms. Additionally, when examining patient preference between treatment regimens after unblinding, no data are presented to fully explain patient choice, such as patient-reported outcomes. However, we do note that IRR frequency among patients after treatment choice was assessed as a possible factor influencing patient preference.

## Discussion

In the ENSEMBLE PLUS substudy, we previously reported similar rates and severity of IRRs between conventional and shorter infusions of OCR at first RD for the primary analyses [9]. The data presented herein included more patients who entered the study after the primary analyses. The results show that the frequency, severity, and symptoms of IRRs were similar between conventional and shorter OCR infusions across all RDs. The primary analysis was descriptive, and no formal hypothesis testing was performed; however, the upper CI of the IRR difference between the two arms (8.2%) can be used to establish the benefit/risk of the shorter infusion, in a similar way as for a non inferiority test. In addition, such a difference should be weighed against the severity of the IRR events.

While the overall frequency of IRRs may seem high (41.6% and 46.2% in the conventional and shorter infusion groups, respectively), we note that the aim of this substudy, to examine IRR frequency, may have resulted in an increase in IRR observations. Additionally, the severity of IRRs for both infusion arms was generally low, with the majority of IRRs across all RDs of Grade 1 or Grade 2; the incidence of Grade 3 IRRs was very low, and there were no Grade 4 or Grade 5 IRRs reported across either infusion group. Furthermore, no correlation was observed between peak OCR serum concentration and IRR incidence. There were three deaths overall, which were all COVID-19–related and all in unvaccinated patients.

Overall, the high number of patients (98%) opting to continue in the main ENSEMBLE study demonstrates that IRRs were tolerable for patients and suggest that the benefits of shorter infusion time outweighed the IRRs experienced. IRR infusion symptoms were similar between conventional and shorter infusion groups. However, differences in IRR symptoms were observed depending on the onset of the IRR (during versus 24 h post-infusion). At first RD, the most frequent IRR symptoms were throat irritation and dysphagia, during the infusion, and headache and fatigue, within 24 h post-infusion. The most common symptomatic treatments used to treat IRRs at first RD were paracetamol, diphenhydramine hydrochloride, and chlorphenamine. Relatively few of the observed IRRs (< 15%) led to intervention in the shorter infusion group, and the median time for shorter infusion remained at 120 min despite some patients having slowed/interrupted infusions; interventions were generally minor with limited effect on the median infusion time. There were no IRR-related discontinuations from OCR treatment, although three patients had IRR-related discontinuations from the substudy in the shorter infusion arm. In general, the results observed in the ITT population were consistent with a per-protocol analysis with an IRR of 100/370 (27.0%) and 108/375 (28.8%) in the conventional and shorter arms, respectively (difference, stratified estimates [95% CI]: 2.0% [− 4.3, 8.3]). A higher proportion of patients with an IRR was observed in the shorter infusion arm; however, closer inspection of these data shows this was largely driven by IRRs at subsequent doses, which had very low patient numbers, potentially resulting in overestimation of differences between treatment arms. The higher rate of IRRs observed in patients in the shorter infusion group receiving the first RD at Dose 4, 5, or 6 was potentially due to an imbalance in the number of patients with prior IRRs before the first RD rather than a difference in infusion procedure. More patients with pre-randomization IRRs of throat irritation (mainly throat pain and dysphagia) were randomized to the shorter infusion group, which could account for the higher rate of IRRs seen in this group at first RD. Furthermore, when looking only at patients without any prior IRR, the rate of IRRs at first RD was similar in both infusion groups (19.6% and 19.1% in the conventional and shorter infusion groups, respectively). This observation suggests that prior IRRs may therefore be an important predictor for further IRRs with OCR treatment. IRRs experienced prior to randomization have previously been suggested to increase the probability of IRRs at a later dose; consistent with this notion, there is strong evidence that a prefirst RD IRR symptom of throat irritation may be a predictive risk factor for the development of throat irritation as an IRR symptom after randomization. However, not all pre-randomization IRR symptoms were predictive of future IRRs; for example, the proportion of patients with headache/migraine as an IRR symptom after randomization was similar regardless of previous medical history for this IRR symptom.

Premedication (which mostly included methylprednisolone or its equivalent, and antihistamine as per the ENSEMBLE main study protocol) was provided prior to infusion; small changes to premedication, such as addition of an antipyretic or adjustments to route and timing, were permitted at all doses following first RD. The proportion of change in premedication among subjects who had an IRR on previous doses was similar between the two arms. Although the safety of shorter OCR infusion is still to be determined in other MS phenotypes, existing data from pivotal phase 3 studies (OPERA and ORATORIO) gave evidence for a similar IRR profile in patients with relapsing MS and primary progressive MS (PPMS) [5]. Safety results of shorter OCR infusion in patients with RRMS, who had a suboptimal response to previous disease-modifying therapies (CHORDS study; NCT02637856) [11], were similar to the present study. A small study of shorter OCR infusion in patients with PPMS also did not demonstrate any signals to indicate phenotype impact on rate or severity of IRRs (SaROD study; NCT03606460) [12].

In general, AEs observed during the ENSEMBLE substudy were consistent with the known safety profile of OCR in patients with RRMS [3–5], and no new safety signals were observed.

## Conclusion

Overall, results from the final analysis of the ENSEMBLE PLUS substudy further demonstrate that OCR infusion time may be reduced from 3.5 h to 2.0 h, without altering the safety profile. The majority of patients chose to either stay on, or move to, the shorter infusion (*n* = 610) after unblinding the study, despite 42% of these patients having experienced IRRs during the blinded period, strongly suggesting that IRRs were tolerable to patients. Most patients (98%) opted to continue OCR treatment in the main ENSEMBLE study at the conclusion of the substudy, further demonstrating that the safety/tolerability profile of OCR is acceptable to patients. Reducing OCR infusion time substantially reduces the administration burden for both patients and site staff, reflected by the general patient preference for shorter infusion. Revised OCR prescribing guidance now permits a shorter infusion time of 2 h, thus reducing infusion site stays and potentially easing patient and hospital staff burden. Additional benefits of improved adherence, time, and cost savings are likely to be observed and may be a focus of future research. While these findings support the use of a shorter infusion protocol for patients with RRMS, further data are required to determine the safety of this protocol in other MS phenotypes.

### Supplementary Information

Below is the link to the electronic supplementary material.

Supplementary file1 (PDF 156 KB)

Supplementary file2 (PDF 181 KB)

Supplementary file3 (PDF 184 KB)

## Data Availability

For up-to-date details on Roche’s Global Policy on the Sharing of Clinical Information and how to request access to related clinical study documents, see here: https://go.roche.com/data_sharing. Anonymized records for individual patients across more than one data source external to Roche cannot, and should not, be linked due to a potential increase in risk of patient re-identification. For eligible studies, qualified researchers may request access to individual patient-level clinical data through a data request platform. At the time of writing this request platform is Vivli: https://vivli.org/ourmember/roche/.
