# Supplementary material for: ENSEMBLE PLUS: final results of shorter ocrelizumab infusion from a randomized controlled trial
Source: J Neurol. 2024 Apr 22;271(7):4348–60. doi: 10.1007/s00415-024-12326-z (PMC11233283; doi:10.1007/s00415-024-12326-z)
Supplement: Supplementary file 1 — Supplementary file1 (PDF 156 KB) [file 415_2024_12326_MOESM1_ESM.pdf]

ENSEMBLE PLUS: final results of shorter ocrelizumab infusion from a randomized controlled trial

Journal of Neurology

Authors: Hans-Peter Hartung, Thomas Berger, Robert A. Bermel, Bruno Brochet, William M. Carroll, Trygve Holmøy, Rana Karabudak, Joep Killestein, Carlos Nos, Francesco Patti, Amy Perrin Ross, Ludo Vanopdenbosch, Timothy Vollmer, Regine Buffels, Monika Garas, Karen Kadner, Marianna Manfrini, Qing Wang, Mark S. Freedman

Corresponding author:
Hans-Peter Hartung
Department of Neurology, UKD, Centre of Neurology and Neuropsychiatry and LVR-Klinikum, Heinrich-Heine University Düsseldorf, Düsseldorf, Germany
Email: hans-peter.hartung@uni-duesseldorf.de

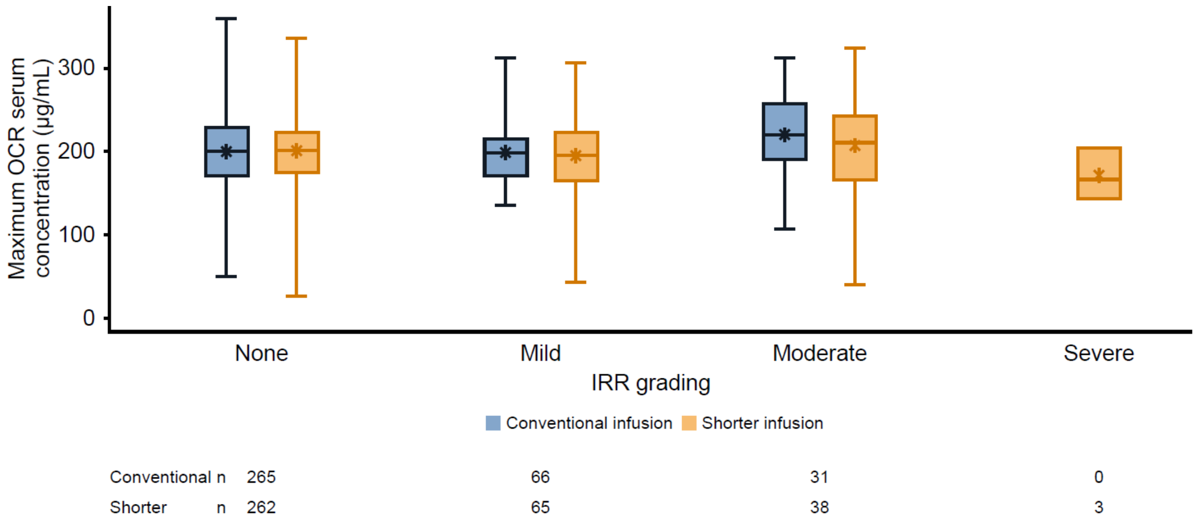

Supplementary Fig. 1 Box plot of maximum OCR serum concentration (µg/mL) vs IRR maximum intensity by 1st RD

*Denotes mean; horizontal line within box denotes median, box length denotes first and third quartiles, and box whiskers denote minimum and maximum. For patients receiving conventional infusion, the maximum OCR serum concentration is measured 30 minutes (±10 minutes) after completion of OCR infusion and for patients receiving shorter infusion 30 minutes (±10 minutes) after the switch. *IRR* infusion-related reaction, *OCR* ocrelizumab, *RD* randomized dose
