# Supplementary material for: ENSEMBLE PLUS: final results of shorter ocrelizumab infusion from a randomized controlled trial
Source: J Neurol. 2024 Apr 22;271(7):4348–60. doi: 10.1007/s00415-024-12326-z (PMC11233283; doi:10.1007/s00415-024-12326-z)
Supplement: Supplementary file 2 — Supplementary file2 (PDF 181 KB) [file 415_2024_12326_MOESM2_ESM.pdf]

**ENSEMBLE PLUS: final results of shorter ocrelizumab infusion from a randomized controlled trial**

**Journal of Neurology**

**Authors: Hans-Peter Hartung, Thomas Berger, Robert A. Bermel, Bruno Brochet, William M. Carroll, Trygve Holmøy, Rana Karabudak, Joep Killestein, Carlos Nos, Francesco Patti, Amy Perrin Ross, Ludo Vanopdenbosch, Timothy Vollmer, Regine Buffels, Monika Garas, Karen Kadner, Marianna Manfrini, Qing Wang, Mark S. Freedman**

**Corresponding author:**
**Hans-Peter Hartung**
**Department of Neurology, UKD, Centre of Neurology and Neuropsychiatry and LVR-Klinikum, Heinrich-Heine University Düsseldorf, Düsseldorf, Germany**
**Email: hans-peter.hartung@uni-duesseldorf.de**

**Supplementary Table 1A** IRR symptoms during first RD (IRR during OCR/saline infusion)

| | Conventional infusion (n=373) | Shorter infusion (n=372) |
|---|---|---|
| **Number (%) of patients with an infusion** | 373 (100) | 372 (100) |
| **Number (%) of patients with any IRR** | 101 (27.1) | 107 (28.8) |
| **Number (%) of patients with any IRR during the OCR/saline infusion** | 44 (43.6) | 65 (60.7) |
| **Respiratory, thoracic, and mediastinal disorders** | 24 (23.8) | 40 (37.4) |
| Throat irritation | 19 (18.8) | 32 (29.9) |
| Oropharyngeal pain | 4 (4.0) | 6 (5.6) |
| Throat tightness | 2 (2.0) | 1 (0.9) |
| Dyspnea | 1 (1.0) | 1 (0.9) |
| Pharyngeal swelling | 0 (0.0) | 2 (1.9) |
| Dry throat | 1 (1.0) | 0 (0.0) |
| Increased upper airway secretion | 0 (0.0) | 1 (0.9) |
| Laryngeal inflammation | 1 (1.0) | 0 (0.0) |
| Nasal congestion | 0 (0.0) | 1 (0.9) |
| Oropharyngeal edema | 0 (0.0) | 1 (0.9) |
| **Gastrointestinal disorders** | 9 (8.9) | 11 (10.3) |
| Dysphagia | 7 (6.9) | 8 (7.5) |
| Nausea | 0 (0.0) | 2 (1.9) |
| Dyspepsia | 1 (1.0) | 0 (0.0) |
| Glossodynia | 0 (0.0) | 1 (0.9) |
| Lip pruritus | 1 (1.0) | 0 (0.0) |
| Odynophagia | 0 (0.0) | 1 (0.9) |
| Oral pain | 0 (0.0) | 1 (0.9) |
| **Skin and subcutaneous tissue disorders** | 7 (6.9) | 10 (9.3) |

| | | |
|---|---|---|
| Rash | 1 (1.0) | 7 (6.5) |
| Pruritus | 3 (3.0) | 3 (2.8) |
| Erythema | 2 (2.0) | 0 (0.0) |
| Rash pruritic | 1 (1.0) | 0 (0.0) |
| **Ear and labyrinth disorders** | 6 (5.9) | 7 (6.5) |
| Ear pruritus | 6 (5.9) | 6 (5.6) |
| Ear discomfort | 0 (0.0) | 1 (0.9) |
| **Nervous system disorders** | 4 (4.0) | 5 (4.7) |
| Headache | 3 (3.0) | 3 (2.8) |
| Burning sensation | 0 (0.0) | 1 (0.9) |
| Sensory disturbance | 0 (0.0) | 1 (0.9) |
| Somnolence | 1 (1.0) | 0 (0.0) |
| **General disorders and administration site conditions** | 6 (5.9) | 2 (1.9) |
| Chest discomfort | 4 (4.0) | 0 (0.0) |
| Fatigue | 1 (1.0) | 1 (0.9) |
| Feeling hot | 0 (0.0) | 1 (0.9) |
| Influenza-like illness | 1 (1.0) | 0 (0.0) |
| **Vascular disorders** | 0 (0.0) | 4 (3.7) |
| Hypotension | 0 (0.0) | 2 (1.9) |
| Hypertension | 0 (0.0) | 1 (0.9) |
| Pallor | 0 (0.0) | 1 (0.9) |
| **Eye disorders** | 1 (1.0) | 1 (0.9) |
| Eye pruritus | 1 (1.0) | 0 (0.0) |
| Lacrimation increased | 0 (0.0) | 1 (0.9) |
| **Investigations** | 1 (1.0) | 1 (0.9) |
| Blood pressure diastolic decreased | 1 (1.0) | 0 (0.0) |
| Blood pressure increased | 0 (0.0) | 1 (0.9) |
| **Cardiac disorders** | 0 (0.0) | 1 (0.9) |
| Bradycardia | 0 (0.0) | 1 (0.9) |
| **Musculoskeletal and connective tissue disorders** | 1 (1.0) | 0 (0.0) |
| Back pain | 1 (1.0) | 0 (0.0) |

**Supplementary Table 1B** IRR symptoms during first RD (IRR with 24 hours after end of OCR/saline infusion)

| | **Conventional infusion (n=373)** | **Shorter infusion (n=372)** |
|---|---|---|
| **Number (%) of patients with any IRR within 24 hours after the end of the OCR/saline infusion** | 68 (67.3) | 54 (50.5) |
| **Nervous system disorders** | 34 (33.7) | 22 (20.6) |
| Headache | 26 (25.7) | 19 (17.8) |
| Dizziness | 4 (4.0) | 1 (0.9) |
| Tremor | 2 (2.0) | 1 (0.9) |
| Paraesthesia | 2 (2.0) | 0 (0.0) |
| Tension headache | 1 (1.0) | 1 (0.9) |
| Disturbance in attention | 1 (1.0) | 0 (0.0) |
| Migraine | 1 (1.0) | 0 (0.0) |
| **General disorders and administration site conditions** | 31 (30.7) | 22 (20.6) |
| Fatigue | 23 (22.8) | 20 (18.7) |
| Pyrexia | 4 (4.0) | 1 (0.9) |
| Chest discomfort | 1 (1.0) | 0 (0.0) |
| Chest pain | 1 (1.0) | 0 (0.0) |
| Feeling hot | 1 (1.0) | 0 (0.0) |
| Feeling jittery | 1 (1.0) | 0 (0.0) |
| Injection site bruising | 1 (1.0) | 0 (0.0) |
| Pain | 1 (1.0) | 0 (0.0) |
| Peripheral swelling | 0 (0.0) | 1 (0.9) |
| Swelling face | 0 (0.0) | 1 (0.9) |
| Thirst | 1 (1.0) | 0 (0.0) |
| **Gastrointestinal disorders** | 9 (8.9) | 11 (10.3) |
| Nausea | 8 (7.9) | 7 (6.5) |
| Diarrhea | 0 (0.0) | 2 (1.9) |
| Abdominal discomfort | 0 (0.0) | 1 (0.9) |
| Faeces soft | 0 (0.0) | 1 (0.9) |
| Flatulence | 1 (1.0) | 0 (0.0) |
| **Respiratory, thoracic, and mediastinal disorders** | 11 (10.9) | 5 (4.7) |
| Oropharyngeal pain | 3 (3.0) | 2 (1.9) |
| Throat irritation | 4 (4.0) | 1 (0.9) |
| Dyspnea | 3 (3.0) | 0 (0.0) |
| Dry throat | 1 (1.0) | 0 (0.0) |
| Dyspnea exertional | 0 (0.0) | 1 (0.9) |
| Nasal congestion | 0 (0.0) | 1 (0.9) |
| Sneezing | 0 (0.0) | 1 (0.9) |
| **Vascular disorders** | 13 (12.9) | 3 (2.8) |

| | | |
|---|---|---|
| Flushing | 9 (8.9) | 3 (2.8) |
| Hot flush | 3 (3.0) | 0 (0.0) |
| Pallor | 1 (1.0) | 0 (0.0) |
| **Musculoskeletal and connective tissue disorders** | 7 (6.9) | 4 (3.7) |
| Pain in extremity | 3 (3.0) | 1 (0.9) |
| Arthralgia | 1 (1.0) | 1 (0.9) |
| Back pain | 0 (0.0) | 2 (1.9) |
| Myalgia | 2 (2.0) | 0 (0.0) |
| Limb discomfort | 0 (0.0) | 1 (0.9) |
| Muscle fatigue | 1 (1.0) | 0 (0.0) |
| **Skin and subcutaneous tissue disorders** | 5 (5.0) | 3 (2.8) |
| Pruritus | 3 (3.0) | 1 (0.9) |
| Rash | 3 (3.0) | 0 (0.0) |
| Erythema | 0 (0.0) | 2 (1.9) |
| **Cardiac disorders** | 2 (2.0) | 4 (3.7) |
| Tachycardia | 1 (1.0) | 3 (2.8) |
| Palpitations | 2 (2.0) | 1 (0.9) |
| **Ear and labyrinth disorders** | 0 (0.0) | 1 (0.9) |
| Vertigo | 0 (0.0) | 1 (0.9) |
| **Infections and infestations** | 1 (1.0) | 0 (0.0) |
| Oral herpes | 1 (1.0) | 0 (0.0) |
| **Injury, poisoning, and procedural complications** | 1 (1.0) | 0 (0.0) |
| Muscle strain | 1 (1.0) | 0 (0.0) |
| **Investigations** | 1 (1.0) | 0 (0.0) |
| Body temperature increased | 1 (1.0) | 0 (0.0) |

Percentages for number of patients with an infusion are based on n, and percentages for number of patients with any IRR are based on number of patients with an infusion. Percentages of patients with symptoms within each time period (e.g., "During OCR/saline Infusion") are based on number of patients with any IRR. Percentages of patients with any symptoms are based on number of patients with any IRR. IRR symptoms are displayed in descending order of frequency of System Organ Class and by preferred term within System Organ Class. If a patient experienced more than one episode of an IRR symptom, then the patient is counted only once for that symptom. If a patient had more than one symptom in a System Organ Class, then the patient is counted only once in that System Organ Class. System Organ Class and preferred terms were defined using MedDRA Version 24.1 thesaurus terms. *IRR* infusion-related reaction, *MedDRA* Medical Dictionary for Regulatory Activities, *OCR* ocrelizumab, *RD* randomized dose
