# Supplementary material for: ENSEMBLE PLUS: final results of shorter ocrelizumab infusion from a randomized controlled trial
Source: J Neurol. 2024 Apr 22;271(7):4348–60. doi: 10.1007/s00415-024-12326-z (PMC11233283; doi:10.1007/s00415-024-12326-z)
Supplement: Supplementary file 3 — Supplementary file3 (PDF 184 KB) [file 415_2024_12326_MOESM3_ESM.pdf]

ENSEMBLE PLUS: final results of shorter ocrelizumab infusion from a randomized controlled trial

Journal of Neurology

Authors: Hans-Peter Hartung, Thomas Berger, Robert A. Bermel, Bruno Brochet, William M. Carroll, Trygve Holmøy, Rana Karabudak, Joep Killestein, Carlos Nos, Francesco Patti, Amy Perrin Ross, Ludo Vanopdenbosch, Timothy Vollmer, Regine Buffels, Monika Garas, Karen Kadner, Marianna Manfrini, Qing Wang, Mark S. Freedman

Corresponding author:
Hans-Peter Hartung
Department of Neurology, UKD, Centre of Neurology and Neuropsychiatry and LVR-Klinikum, Heinrich-Heine University Düsseldorf, Düsseldorf, Germany
Email: hans-peter.hartung@uni-duesseldorf.de

Supplementary Table 2 Proportion of patients with an IRR following each RD and subsequent doses

	**Conventional infusion (n=373)**	**Shorter infusion (n=372)**
**1st RD**		
**Overall**		
Number (%) of patients with an infusion	373 (100)	372 (100)
Number (%) of patients with any IRR	101 (27.1)	107 (28.8)
**Dose 2**		
Number (%) of patients with an infusion	269 (72.1)	270 (72.6)
Number (%) of patients with any IRR	85 (31.6)	83 (30.7)
**Dose 3**		
Number (%) of patients with an infusion	24 (6.4)	22 (5.9)
Number (%) of patients with any IRR	3 (12.5)	2 (9.1)
**Dose 4**		
Number (%) of patients with an infusion	61 (16.4)	59 (15.9)
Number (%) of patients with any IRR	9 (14.8)	17 (28.8)
**Dose 5**		
Number (%) of patients with an infusion	19 (5.1)	20 (5.4)
Number (%) of patients with any IRR	4 (21.1)	5 (25.0)
**Dose 6**		
Number (%) of patients with an infusion	0 (0.0)	1 (0.3)
Number (%) of patients with any IRR	0 (0.0)	0 (0.0)
**2nd RD**		
**Overall**		
Number (%) of patients with an infusion	367 (98.4)	355 (95.4)
Number (%) of patients with any IRR	84 (22.9)	96 (27.0)
**Dose 3**		
Number (%) of patients with an infusion	264 (70.8)	257(69.1)
Number (%) of patients with any IRR	67 (25.4)	73 (28.4)
**Dose 4**		
Number (%) of patients with an infusion	23 (6.2)	22 (5.9)
Number (%) of patients with any IRR	3 (13.0)	3 (13.6)

**Dose 5**		
Number (%) of patients with an infusion	61 (16.4)	57 (15.3)
Number (%) of patients with any IRR	12 (19.7)	18 (31.6)
**Dose 6**		
Number (%) of patients with an infusion	19 (5.1)	18 (4.8)
Number (%) of patients with any IRR	2 (10.5)	2 (11.1)
**Dose 7**		
Number (%) of patients with an infusion	0 (0.0)	1 (0.3%)
Number (%) of patients with any IRR	0 (0.0)	0 (0.0)
**3rd RD**		
**Overall**		
Number (%) of patients with an infusion	305 (81.8)	300 (80.6)
Number (%) of patients with any IRR	62 (20.3)	82 (27.3)
**Dose 4**		
Number (%) of patients with an infusion	210 (56.3)	212 (57.0)
Number (%) of patients with any IRR	51 (24.3)	59 (27.8)
**Dose 5**		
Number (%) of patients with an infusion	23 (6.2)	22 (5.9)
Number (%) of patients with any IRR	3 (13.0)	5 (22.7)
**Dose 6**		
Number (%) of patients with an infusion	58 (15.5)	52 (14.0)
Number (%) of patients with any IRR	7 (12.1)	15 (28.8)
**Dose 7**		
Number (%) of patients with an infusion	14 (3.8)	14 (3.8)
Number (%) of patients with any IRR	1 (7.1)	3 (21.4)
**Dose 8**		
Number (%) of patients with an infusion	0 (0.0)	0 (0.0)
Number (%) of patients with any IRR	0 (0.0)	0 (0.0)
**4th RD**		
**Overall**		
Number (%) of patients with an infusion	147 (39.4)	136 (36.6)
Number (%) of patients with any IRR	14 (9.5)	17 (12.5)
**Dose 5**		
Number (%) of patients with an infusion	81 (21.7)	70 (18.8)
Number (%) of patients with any IRR	7 (8.6)	7 (10.0)
**Dose 6**		
Number (%) of patients with an infusion	22 (5.9)	22 (5.9)
Number (%) of patients with any IRR	1 (4.5)	3 (13.6)
**Dose 7**		
Number (%) of patients with an infusion	36 (9.7)	36 (9.7)
Number (%) of patients with any IRR	5 (13.9)	7 (19.4)
**Dose 8**		
Number (%) of patients with an infusion	8 (2.1)	8 (2.2)
Number (%) of patients with any IRR	1 (12.5)	0 (0.0)
**5th RD**		
**Overall**		
Number (%) of patients with an infusion	23 (6.2)	21 (5.6)
Number (%) of patients with any IRR	1 (4.3)	3 (14.3)
**Dose 6**		
Number (%) of patients with an infusion	10 (2.7)	5 (1.3)

Number (%) of patients with any IRR	0 (0.0)	0 (0.0)
**Dose 7**		
Number (%) of patients with an infusion	10 (2.7)	9 (2.4)
Number (%) of patients with any IRR	0 (0.0)	2 (22.2)
**Dose 8**		
Number (%) of patients with an infusion	3 (0.8)	7 (1.9)
Number (%) of patients with any IRR	1 (33.3)	1 (14.3)
**6th RD**		
**Overall**		
Number (%) of patients with an infusion	6 (1.6)	4 (1.1)
Number (%) of patients with any IRR	0 (0.0)	0 (0.0)
**Dose 7**		
Number (%) of patients with an infusion	3 (0.8)	1 (0.3)
Number (%) of patients with any IRR	0 (0.0)	0 (0.0)
**Dose 8**		
Number (%) of patients with an infusion	3 (0.8)	3 (0.8)
Number (%) of patients with any IRR	0 (0.0)	0 (0.0)

Percentages for number of patients with an infusion are based on n, and percentages for number of patients with any IRR are based on number of patients with an infusion. *IRR* infusion-related reaction, *RD* randomized dose
